# Development and Validation of a 7-Gene Prognostic Signature to Improve Survival Prediction in Pancreatic Ductal Adenocarcinoma

**DOI:** 10.3389/fmolb.2021.676291

**Published:** 2021-05-21

**Authors:** Zengyu Feng, Hao Qian, Kexian Li, Jianyao Lou, Yulian Wu, Chenghong Peng

**Affiliations:** ^1^ Department of General Surgery, Pancreatic Disease Center, Ruijin Hospital, Shanghai Jiao Tong University School of Medicine, Shanghai, China; ^2^ Research Institute of Pancreatic Diseases, Shanghai Jiao Tong University School of Medicine, Shanghai, China; ^3^ Department of General Surgery, The Second Affiliated Hospital, School of Medicine, Zhejiang University, Hangzhou, China

**Keywords:** pancreatic ductal adenocarcinoma, risk score, overall survival, disease-free survival, prognostic signature, immune cell infiltration

## Abstract

**Background:** Previous prognostic signatures of pancreatic ductal adenocarcinoma (PDAC) are mainly constructed to predict the overall survival (OS), and their predictive accuracy needs to be improved. Gene signatures that efficaciously predict both OS and disease-free survival (DFS) are of great clinical significance but are rarely reported.

**Methods:** Univariate Cox regression analysis was adopted to screen common genes that were significantly associated with both OS and DFS in three independent cohorts. Multivariate Cox regression analysis was subsequently performed on the identified genes to determine an optimal gene signature in the MTAB-6134 training cohort. The Kaplan–Meier (K-M), calibration, and receiver operating characteristic (ROC) curves were employed to assess the predictive accuracy. Biological process and pathway enrichment analyses were conducted to elucidate the biological role of this signature.

**Results:** Multivariate Cox regression analysis determined a 7-gene signature that contained ASPH, DDX10, NR0B2, BLOC1S3, FAM83A, SLAMF6, and PPM1H. The signature had the ability to stratify PDAC patients with different OS and DFS, both in the training and validation cohorts. ROC curves confirmed the moderate predictive accuracy of this signature. Mechanically, the signature was related to multiple cancer-related pathways.

**Conclusion:** A novel OS and DFS prediction model was constructed in PDAC with multi-cohort and cross-platform compatibility. This signature might foster individualized therapy and appropriate management of PDAC patients.

## Introduction

Pancreatic ductal adenocarcinoma is an insidious and aggressive malignancy with a 5-year survival rate not exceeding 10% (Siegel et al., 2021; Garrido-Laguna and Hidalgo, 2015). Unfortunately, its incidence and mortality rates continue to increase, especially among women and people aged 50 years and over (Huang et al., 2021). Owing to the deep-seated location of the pancreas and the lack of available screening approaches of PDAC, most patients are diagnosed at an advanced, unresectable stage (Ducreux et al., 2015; Gillen et al., 2010), leading to a dismal prognosis (De La Cruz et al., 2014). Even after R0 resection, the most effective treatment for cure (Kamisawa et al., 2016), most patients will develop local recurrence or distant metastases despite adjuvant treatments, impairing a dramatic improvement of patient outcomes by surgical resection (Ghaneh et al., 2019; Strobel et al., 2017; Tummers et al., 2019). Therefore, novel identification of reliable biomarkers and predictive models is of significant need for PDAC patients, which will help to guide appropriate therapy and tailor postoperative surveillance in clinical management.

Accurate prediction of patient prognosis in PDAC has been the subject of many studies. Traditional clinicopathological features, including but not limited to tumor size (Ansari et al., 2017), resection margin status (Maeda et al., 2020), histological grade (Macías et al., 2018), and lymph node metastasis (Sho et al., 2015), have been investigated in previous studies. Recently, with the remarkable progress in bioinformatics and high-throughput sequencing technology, gene expression signatures considering the genetic and genomic differences of patients have emerged as a practical tool for survival assessment in human cancers (Yu et al., 2019; Supplitt et al., 2021; Doultsinos and Mills, 2021; Ahluwalia et al., 2021). In the context of PDAC, multiple prognostic gene models have been established with robust predictive performance (Luo et al., 2021; Gu et al., 2021; Xu et al., 2021a). However, most of these models are constructed to predict the overall survival (OS), and few predict disease-free survival (DFS). Given that postoperative recurrence is a feature of PDAC and it results in a poor prognosis, DFS prediction is of equivalent significance. Thus, development of a robust signature for both OS and DFS prediction is a laudable attempt.

To the best of our knowledge, only two previous gene signatures have the capability to predict both OS and DFS (Kim et al., 2019; Feng et al., 2020), and their predictive accuracy remains to be improved. In the current study, we identified a credible 7-gene signature for both OS and DFS prediction with moderate accuracy and cross-cohort compatibility. The area under the curve (AUC) values of this signature for survival prediction were no less than 0.7 in three independent cohorts. The relationship between gene signature, immune cell infiltration, and therapeutic effects was also investigated in this study.

## Materials and Methods

### PDAC Cohorts

Three public PDAC cohorts with both clinical data and gene expression data including MTAB-6134 (N = 288), PACA-CA (N = 181), and TCGA (N = 141) were adopted in this study. Among them, the MTAB-6134 cohort was used as the training set, while PACA-CA and TCGA cohorts were used for external validation. Information about OS and DFS events and time was available from all of these three cohorts. Expression profiles and clinical data of MTAB-6134 cohort were downloaded from the ArrayExpress database (https://www.ebi.ac.uk/arrayexpress/). Normalized RNA-sequencing (RNA-seq) data and clinical data of the PACA-CA cohort were retrieved and downloaded from the International *Cancer* Genome Consortium (ICGC, https://icgc.org/) database. Processed RNA-seq data and clinical information of the TCGA cohort were obtained from the TCGA hub at UCSC Xena (https://tcga.xenahubs.net). Samples with an OS or DFS less than one month were excluded for survival analyses. Information regarding chemotherapy was provided in PACA-CA and TCGA cohorts. Patients whose response to chemotherapy is “clinical progressive disease” or “stable disease” were defined as chemotherapy-resistant, while patients whose response to chemotherapy is “complete response” or “partial response” were defined as chemotherapy-sensitive. In addition, MTAB-6690, a cohort that contained gene expression profiles of 108 PDAC samples and 70 normal samples, was downloaded from the ArrayExpress database.

### Construction of the 7-Gene Signature

To identify candidate genes for model construction, we initially applied univariate Cox regression analysis to screen genes associated with both OS and DFS through the Venn diagram (https://bioinfogp.cnb.csic.es/tools/venny/) in each cohort. Consistently, survival-related genes in these three cohorts were identified and subsequently submitted to multivariate Cox regression analysis using OS events and time in order to determine an optimal signature in the training MTAB-6134 cohort. Based on the gene expression values and corresponding coefficients, the risk score of each sample was calculated as follows: risk score = (coefficient 1 * expression value of gene 1) + (coefficient 2 * expression value of gene 2) + ... + (coefficient N * expression value of gene N).

### Prognostic Validation of the 7-Gene Signature

Patients in each cohort were divided into low- and high-risk groups based on the medium value of the risk score. The Kaplan–Meier (K-M) survival curves and calibration curves were used to evaluate the predictive performance of this signature. Receiver operating characteristic (ROC) curves were utilized to compare the predictive accuracy of the gene signature and clinical features.

### Estimation of Tumor Immune Infiltrates

The deconvolution algorithm CIBERSORT (Chen et al., 2018) was used to estimate the relative proportions of 22 different immune cell infiltrates. The number of permutations was set to 1,000, and a *p*-value < 0.05 was considered as successful.

### Functional Annotation and Pathway Enrichment of the 7-Gene Signature

To shed light on high-risk score–resulted unfavorable prognosis, we performed the Pearson correlation analysis to identify correlated genes with risk scores in the MTAB-6134 training cohort. According to the correlation coefficients, the top 1,000 positively and negatively correlated genes were submitted to Gene Ontology-Biological Process (GO-BP) analysis and the Kyoto Encyclopedia of Genes and Genomes (KEGG) pathway enrichment analysis on the DAVID online Web site (Huang et al., 2007), respectively.

### Statistical Analysis

Statistical analysis and graphical work were finished in the R environment (version 3.5.2). Cox regression analyses were performed by the “survival” package and visualized by the “forestplot” package. K-M survival curves with log-rank tests were plotted by the “survminer” package. The ROC curves were depicted by the “survivalROC” package. Boxplots were generated from the “ggpubr” package. Calibration curves were derived from the “rms” package. *p* < 0.05 was considered significant.

## Results

### Construction of the 7-Gene Signature

**FIGURE 1:**
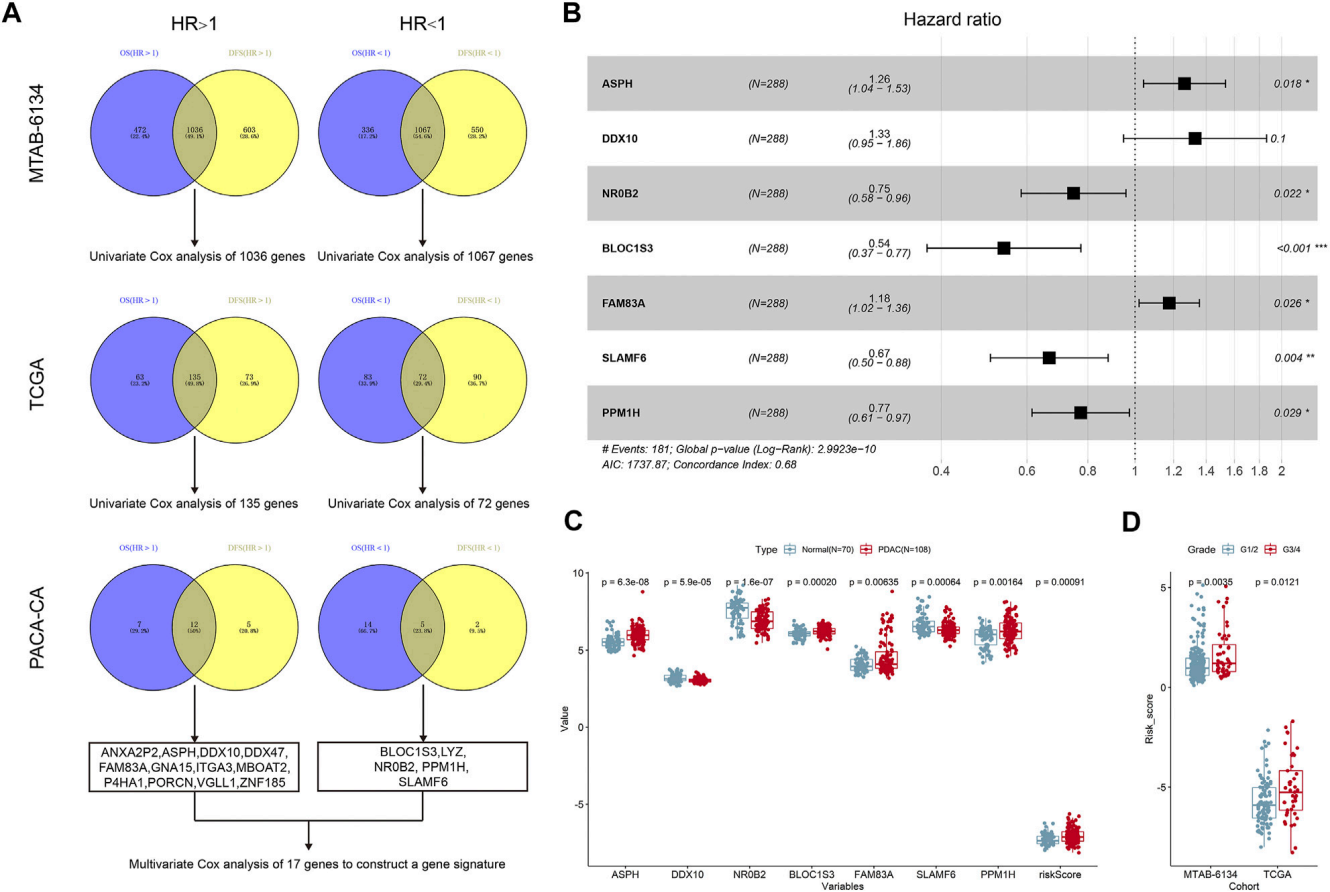
Construction of the 7-gene signature. **(A)** The research workflow of model construction. **(B)** Forest plot of the seven genes. **(C)** Expression values of seven selected genes and risk scores in normal samples and PDAC samples based on the microarray data of MTAB-6690. **(D)** Distribution of risk scores in different histological grades in MTAB-6134 and TCGA samples.

The research workflow of model construction is illustrated in Figure 1A. Univariate Cox analysis and Venn diagram identified 1,036 risky genes (hazard ratio >1) and 1,067 protective genes (hazard ratio <1) that were associated with both OS and DFS in the MTAB-6134 training cohort. These genes were further screened in the PACA-CA cohort and TCGA cohort by the same method, respectively. Eventually, 12 credible risky indicators and five protective indicators were identified. These 17 genes were incorporated in the stepwise multivariate Cox hazard ratio regression to select the best model for predicting OS of PDAC patients in the MTAB-6134 cohort. Multivariate Cox analysis resulted in an optimal 7-gene signature containing ASPH, DDX10, NR0B2, BLOC1S3, FAM83A, SLAMF6, and PPM1H (Figure 1B). The PPI (protein–protein interaction) network was constructed using the STRING database (http://www.string-db.org/) to investigate the interactions of these seven genes. As illustrated in Supplementary Figure S1, there is no interaction between these proteins. According to the expression values and corresponding coefficients of the seven genes derived from multivariate Cox regression analysis, we constructed a risk-score formula: risk score = 0.234165*expression value of ASPH +0.284159*expression value of DDX10-0.29051*expression value of NR0B2-0.61974*expression value of BLOC1S3 + 0. The 161992*expression value of FAM83A-0.40561*expression value of SLAMF6-0.25676*expression value of PPM1H. The risk scores of PDAC patients were significantly higher than those of normal patients, indicating diagnostic potential of the signature (Figure 1C). Moreover, the risk scores were markedly elevated in patients with a high histological grade in MTAB-6134 and TCGA cohorts, suggesting that the 7-gene signature was associated with tumor malignancy (Figure 1D).

### Prognostic Performance of the 7-Gene Signature for OS Prediction

**FIGURE 2:**
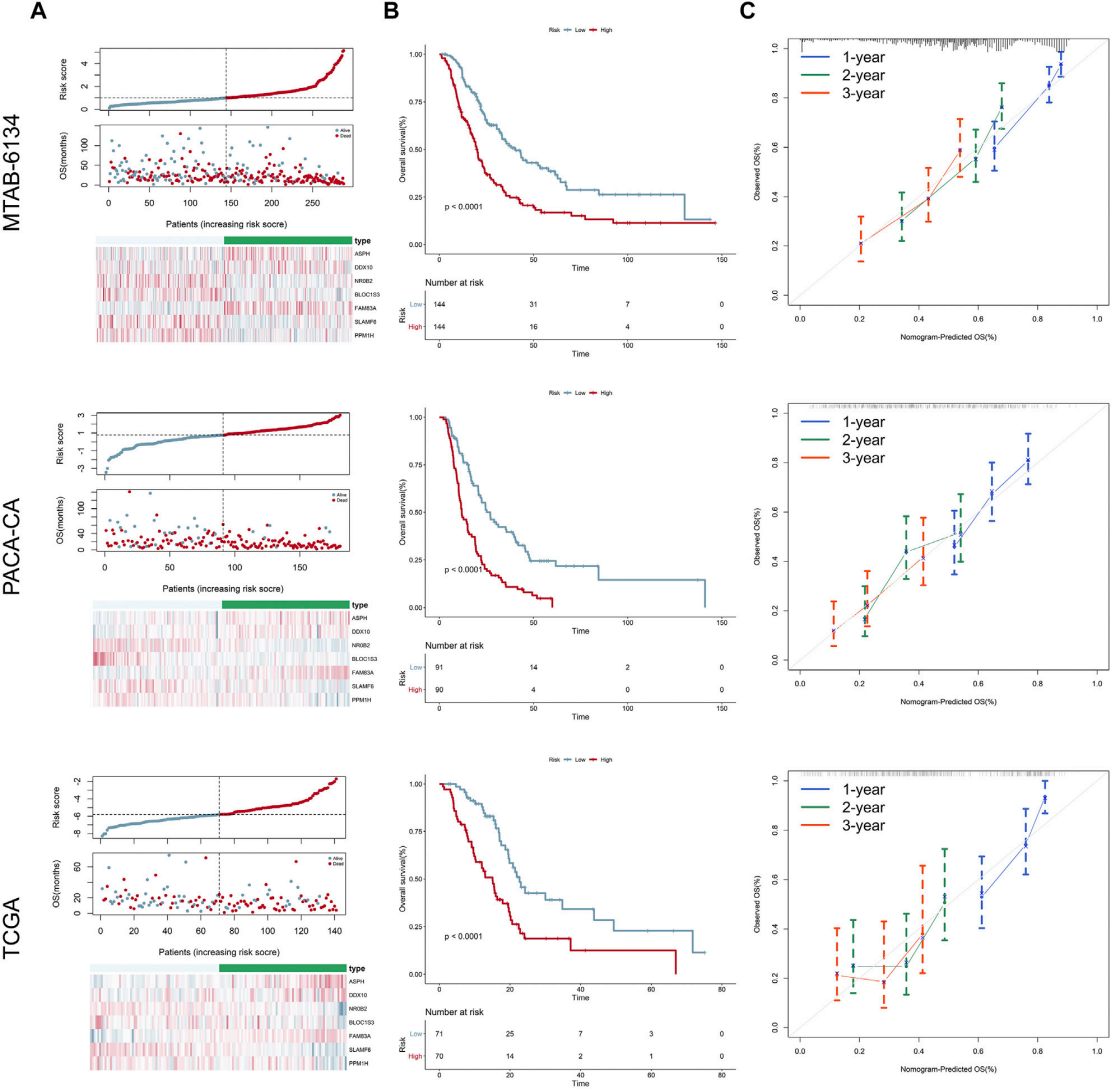
Prognostic performance of the 7-gene signature for OS prediction. **(A)** From top to bottom are the risk score distribution, survival status distribution, and heatmap of seven genes in three independent cohorts, respectively. **(B)** K-M curves estimating the OS difference between low- and high-risk groups in three independent cohorts, respectively. **(C)** Calibration curves of the signature in three independent cohorts. Calibration curves represent the relationship between observed (the data markers represent the mean, and the error bars represent the 95% CI) and predicted (gray line) OS using the 7-gene risk score.

Distribution of the risk scores, survival status, and expression level of the seven genes in three independent cohorts is illustrated in Figure 2A. The results showed that patients in the high-risk group had higher mortality rates. K-M survival curves demonstrated that patients in the low-risk group had a significantly longer OS in three independent cohorts (Figure 2B). Calibration curves suggested that the predicted survival probabilities by the signature were in good agreement with the observed survival probabilities (Figure 2C).

### Predictive Accuracy of the 7-Gene Signature and Clinical Predators for OS

**FIGURE 3:**
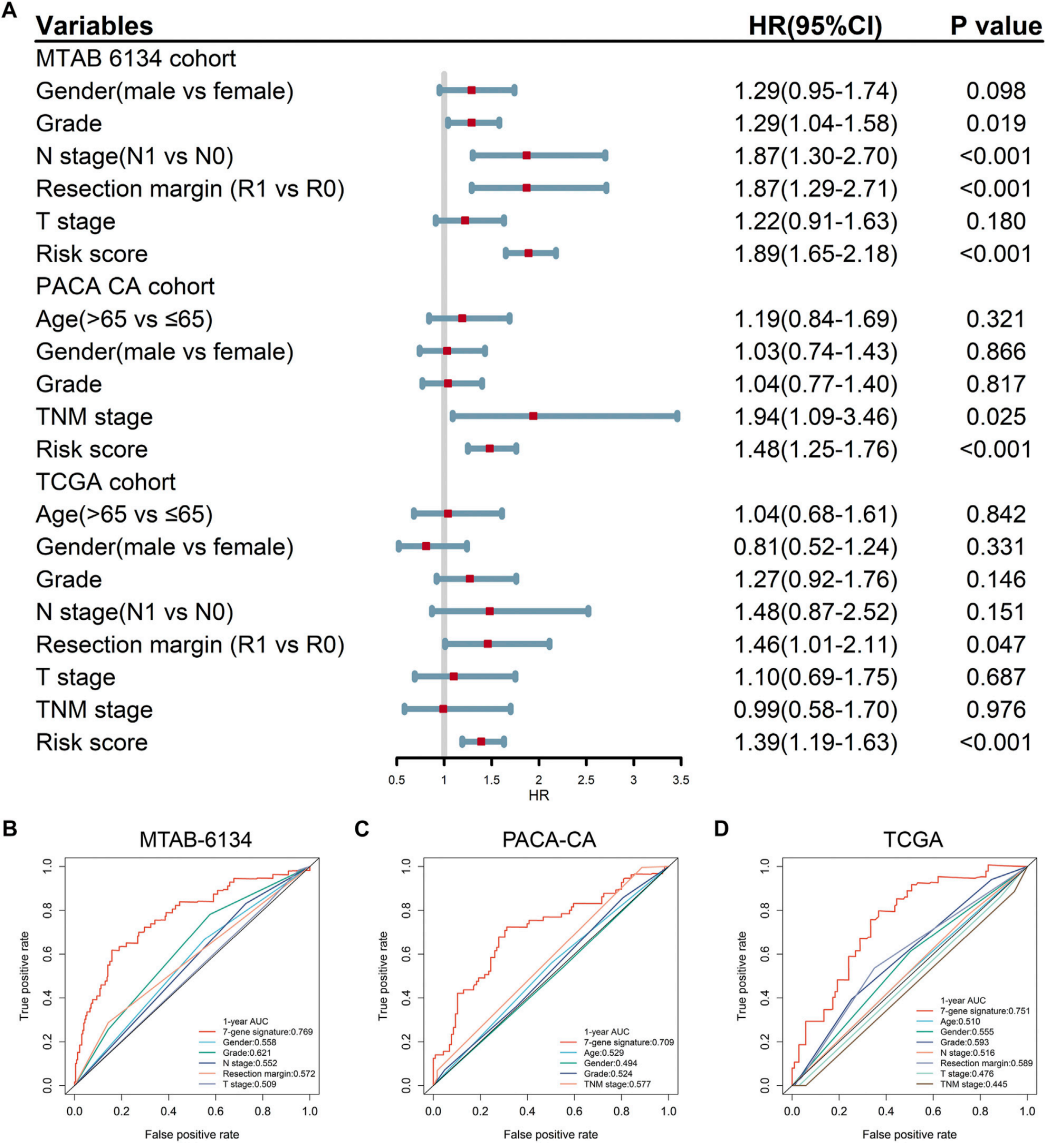
Predictive accuracy of the 7-gene signature and clinical predictors for OS. **(A)** Univariate Cox regression analysis of the gene signature and clinical features for OS. **(B–D)** ROC curves of the risk signature and clinical features for the 1-year OS prediction in MTAB-6134 **(B)**, PACA-CA **(C)**, and TCGA **(D)** cohorts, respectively.

Univariate Cox regression analysis proved that the proposed gene signature and several clinical features were independent risk factors for OS in three cohorts (Figure 3A). In order to clarify whether our signature could provide improved survival prediction, we conducted ROC analyses. As shown in Figures 3B–D, the AUC values of this signature were 0.769, 0.709, and 0.751 in MTAB-6134, PACA-CA, and TCGA cohorts, respectively, which were higher than those of clinical predictors. These findings demonstrated that the 7-gene signature outperformed clinical predictors in predicting OS.

### Prognostic Performance of the 7-Gene Signature for DFS Prediction

**FIGURE 4:**
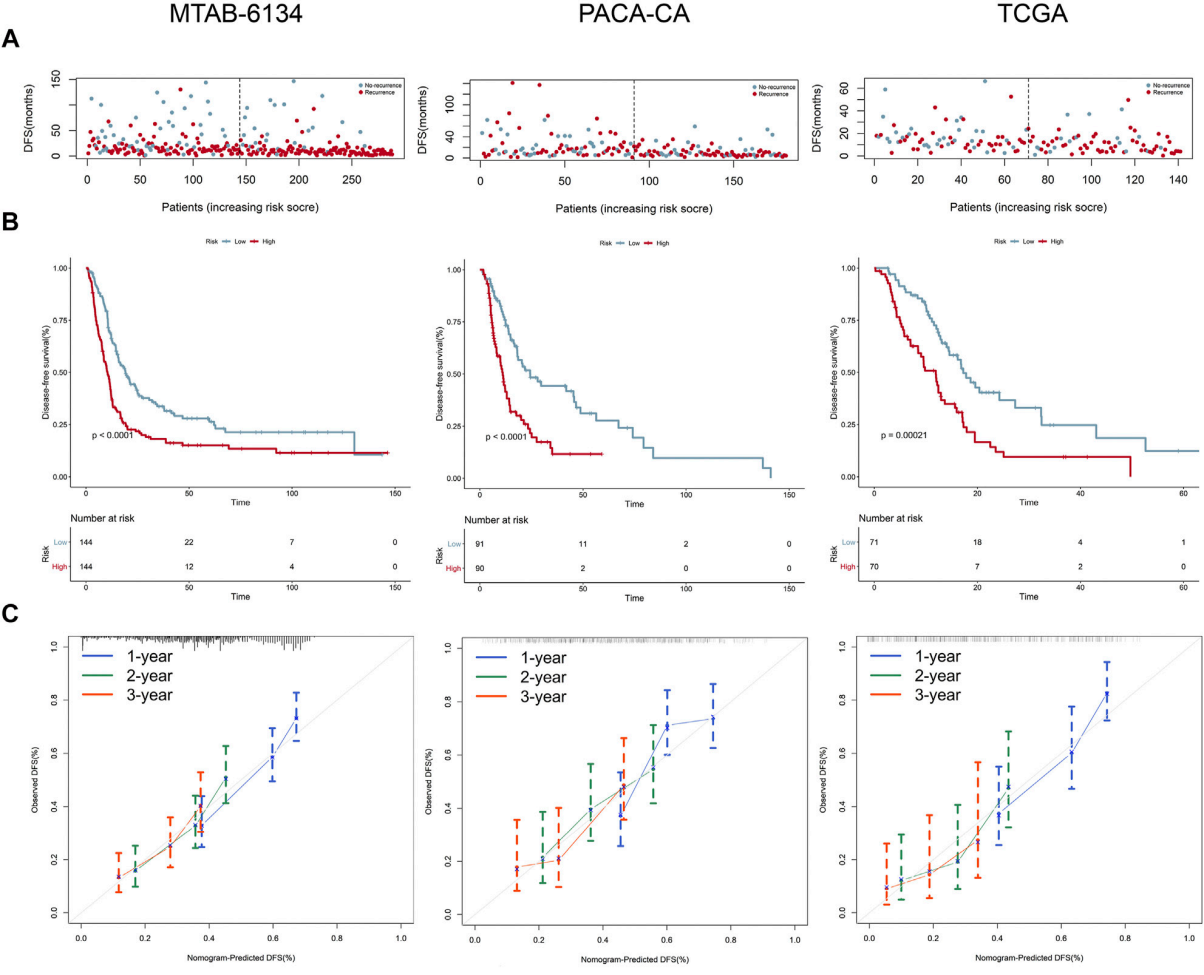
Prognostic performance of the 7-gene signature for the DFS prediction. **(A–C)** From top to bottom are patients’ survival overview, K-M survival curve, and calibration curve in MTAB-6134 **(A)**, PACA-CA **(B),** and TCGA **(C)** cohorts, respectively. Calibration curves represent the relationship between observed (the data markers represent the mean, and the error bars represent the 95% CI) and predicted (gray line) DFS using the 7-gene risk score.

Figure 4A shows the distribution of the DFS event and time in MTAB-6134, PACA-CA, and TCGA cohorts. The results illustrated that patients in the low-risk group had remarkably lower recurrence rates and a significantly longer DFS. K-M survival curves indicated that the high-risk group had a significantly shorter DFS than the low-risk group in all of the three cohorts (*p* < 0.05, Figure 4B). Calibration curves indicated that the predicted DFS was in good accordance with the observed DFS in three independent cohorts (Figure 4C).

### Predictive Accuracy of the 7-Gene Signature and Clinical Predictors for DFS

**FIGURE 5:**
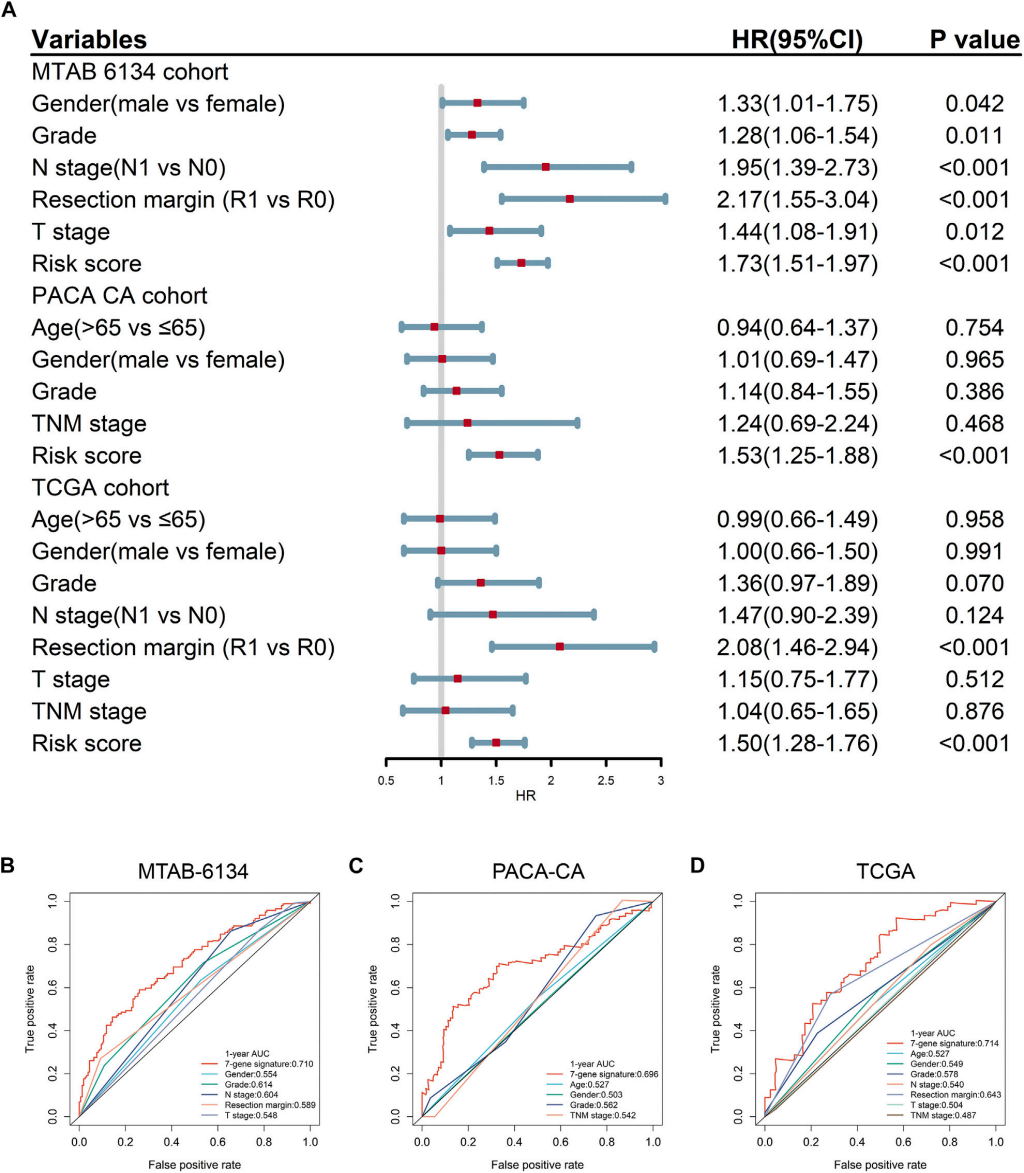
Predictive accuracy of the 7-gene signature and clinical predictors for DFS. **(A)** Univariate Cox regression analysis of gene signature and clinical indicators for DFS. **(B–D)** ROC curves of the risk signature and clinical indicators for the 1-year DFS prediction in MTAB-6134 **(B)**, PACA-CA **(C)**, and TCGA **(D)** cohorts, respectively.

Univariate Cox regression analysis confirmed that the 7-gene signature and multiple clinical indicators were closely related to DFS in three cohorts (Figure 5A). ROC curves showed that the AUC values of this signature for DFS prediction were 0.710, 0.696, and 0.714 in MTAB-6134, PACA-CA, and TCGA cohorts, respectively, which were superior to clinical predictors (Figures 5B–D).

### Relationship Between the 7-Gene Signature and Response to Adjuvant Chemotherapy

**FIGURE 6:**
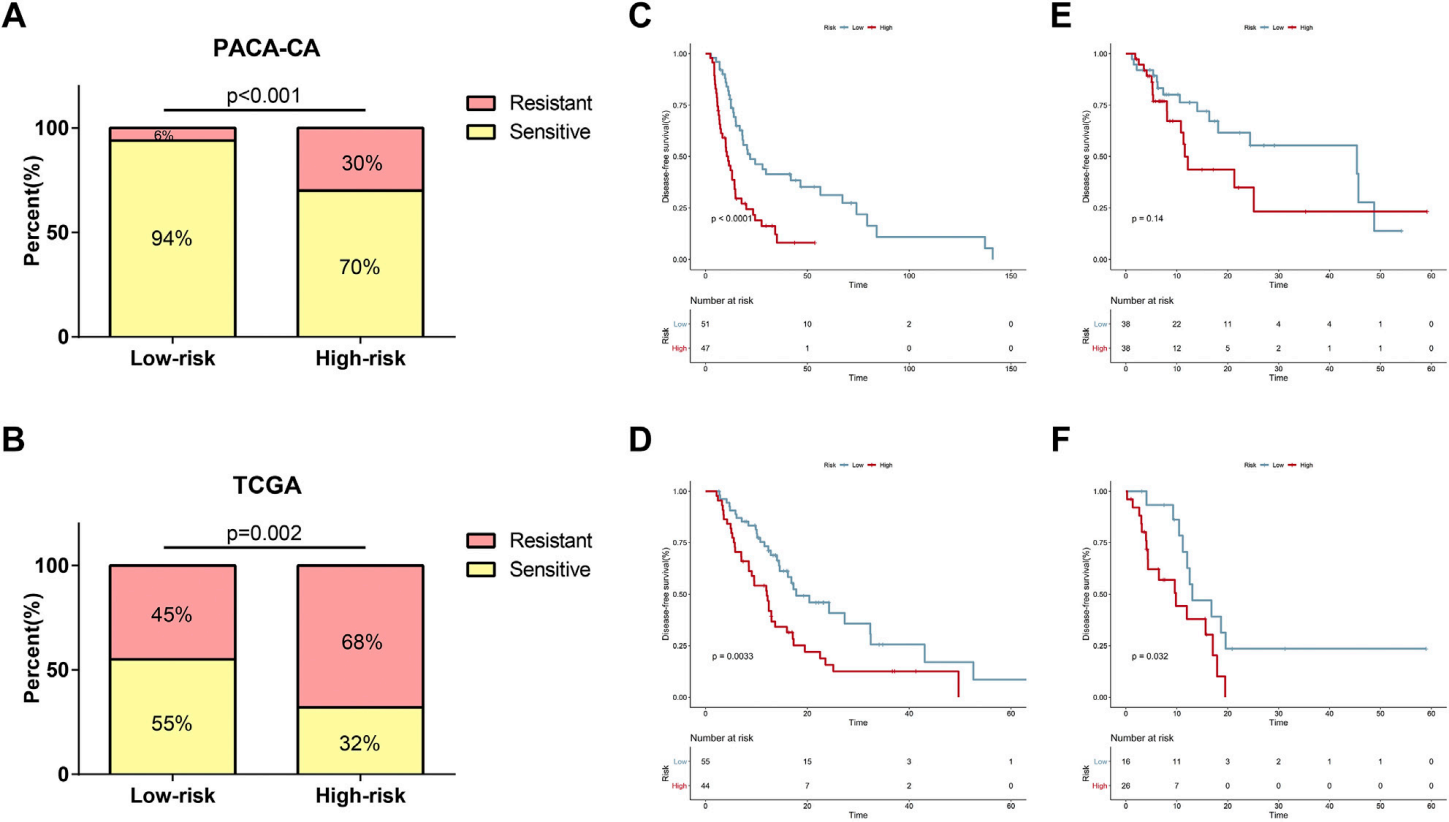
Relationship between the risk signature and response to adjuvant chemotherapy. **(A,B)** Relationship of risk score and chemotherapy sensitivity in the PACA-CA cohort **(A)** and TCGA cohort **(B)**. **(C,D)** K-M curves for the risk signature in patients receiving adjuvant chemotherapy in the PACA-CA cohort **(C)** and TCGA cohort **(D)**. **(E,F)** K-M curves for the risk signature in patients not receiving adjuvant chemotherapy in the PACA-CA cohort **(E)** and TCGA cohort **(F)**.

Currently, adjuvant chemotherapy is administered empirically, and the individual survival benefit of this approach is still questionable in PDAC (Kleeff et al., 2016). Thus, we wondered whether the 7-gene signature could precisely predict chemotherapy sensitivity and provide references for clinical practice. As shown in Figure 6A, patients in the low-risk group had significantly higher response rates to adjuvant chemotherapy than patients in the high-risk group in the PACA-CA cohort (94 vs 70%, *p* < 0.001). A similar trend was seen in the TCGA cohort (55 vs 32%, *p* = 0.001, Figure 6B). In patients who received adjuvant chemotherapy, the proposed signature could efficaciously capture the DFS differences between low-risk and high-risk groups in both cohorts (Figures 6C,D). However, the predictive power of the model was weakened when applied to patients who had not received adjuvant chemotherapy. As shown in Figure 6E, DFS difference between high-risk and low-risk groups of this subset of patients was not significant in the PACA-CA cohort (*p* = 0.14). In the TCGA cohort, DFS difference was also not strongly trustworthy (*p* = 0.034, Figure 6F).

### Relationship Between the 7-Gene Signature and Immune Cell Infiltration

**FIGURE 7:**
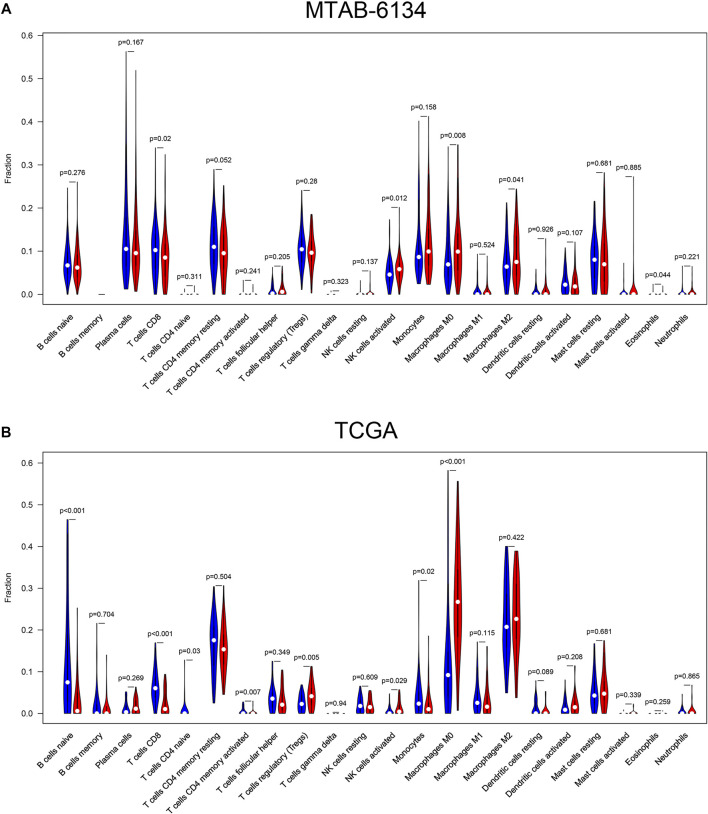
Correlations between the risk signature and different immune cell infiltration in the MTAB-6134 cohort **(A)** and TCGA cohort **(B)**. The red color represented high-risk group, while the blue color represented low-risk group.

The level of immune cell infiltration is closely related to the clinical efficiency of immunotherapy and the prognosis of PDAC patients (Zheng et al., 2013). Therefore, we explored the relationship between this signature and immune cell infiltration and inquired into the potential of our signature as a reliable predictor of immunotherapy response. In cases of the MTAB-6134 cohort, the abundance of macrophage M0, macrophage M2, and activated NK cells was significantly elevated in the high-risk patients, while CD8^+^ T cell infiltration was remarkably decreased in the high-risk patients (Figure 7A). In the case of the TCGA cohort, the abundance of regulatory T cells, activated NK cells, and macrophage M0 was significantly elevated in the high-risk patients, while naïve B cells, CD8^+^ T cell, naïve CD4^+^ T cells, activated memory CD4^+^ T cells, and monocyte infiltration were remarkably decreased in the high-risk patients (Figure 7B).

### Biological Process and Pathway Analyses of the 7-Gene Signature

**FIGURE 8:**
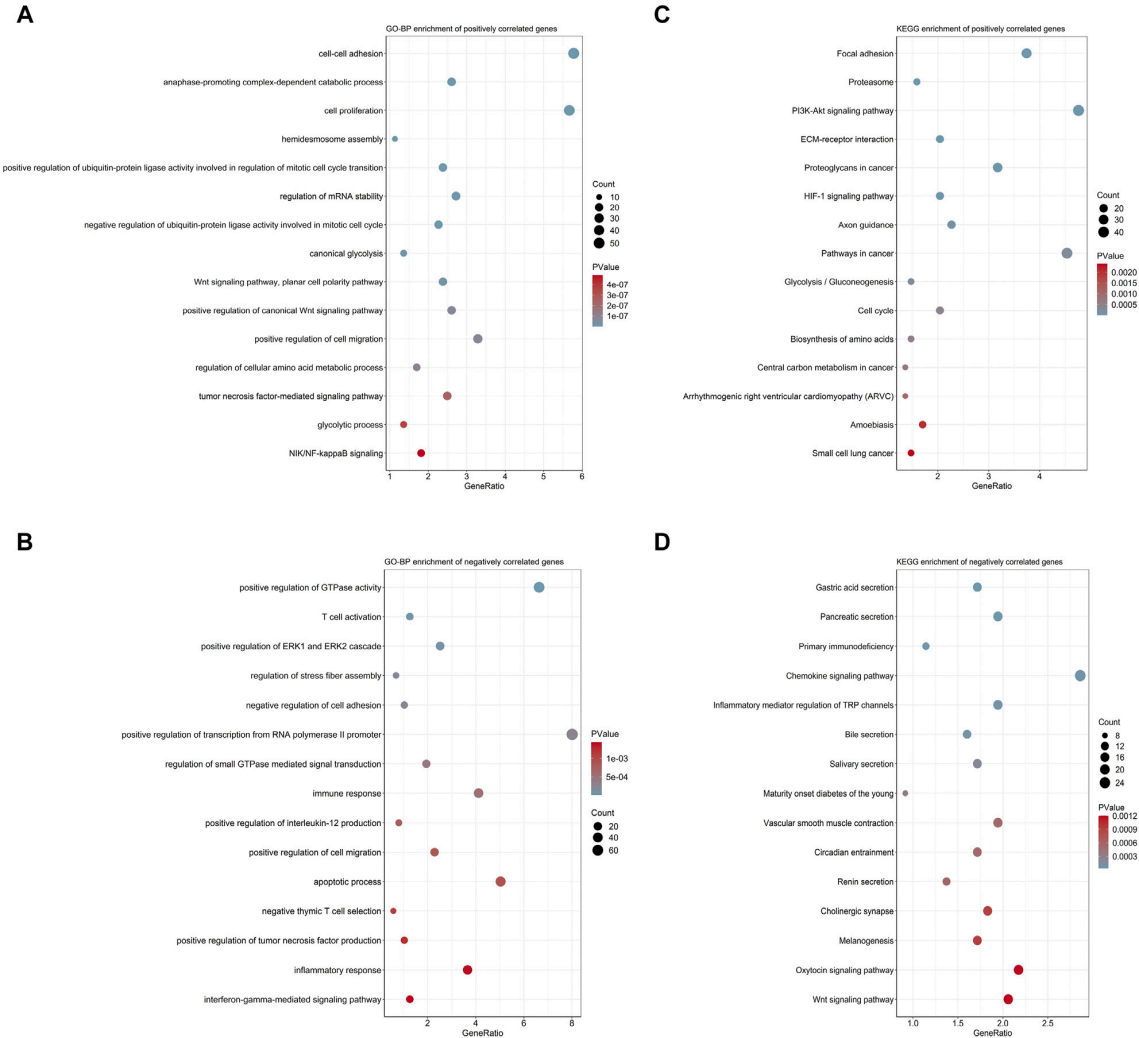
Biological process and pathway analyses of the 7-gene signature. **(A)** Top 15 GO-BP terms of genes positively correlated with risk scores. **(B)** Top 15 GO-BP terms of genes negatively correlated with risk scores. **(C)** Top 15 KEGG terms of genes positively correlated with risk scores. **(D)** Top 15 KEGG terms of genes negatively correlated with risk scores.

With the purpose to preliminarily illuminate how the risk signature affected patient prognosis, chemotherapeutic response, and immune cell infiltration, we performed functional annotation and pathway enrichment analyses on genes correlated with risk scores in the MTAB-6134 training cohort. For biological processes, positively correlated genes were primarily involved in cell proliferation, cell migration, and glycolysis (Figure 8A), while negatively correlated genes were mainly related to T-cell activation, immune response, and apoptotic process (Figure 8B). For KEGG pathway enrichment, genes with positive correlation were chiefly associated with the PI3K-AKT signaling pathway, HIF-1 signaling pathway, glycolysis, and cell cycle (Figure 8C), while genes with negative correlation were principally enriched in primary immunodeficiency and the chemokine signaling pathway (Figure 8D).

## Discussion

PDAC is a fatal disease featured with high molecular heterogeneity. The current prognosis assessment of PDAC patients is mainly dependent on the TNM (tumor, node, and metastasis) staging system. However, it is not adequate for individual survival prediction, especially in patients at the same stage (van Roessel et al., 2018). This intra-stage discrepancy is due to tumor heterogeneity (Juiz et al., 2019). As cancer treatment has entered into the area of precision medicine, overcoming molecular heterogeneity has become a hallmark in cancer research (McGranahan and Swanton, 2017). Hence, prognostic gene signatures that translate the increased understanding of tumor genetic and genomic alternations into clinical application are urgently needed. In this study, we developed and validated a 7-gene signature with powerful prognostic performance for both OS and DFS prediction in PDAC. These seven genes were not overlapped to other prognostic gene signatures for PDAC.

In the process of signature construction, we initially identified and overlapped genes associated with OS and DFS in three independent large cohorts. A total of 17 genes were screened, and multivariate Cox regression analysis determined an optimal 7-gene signature for OS prediction in the MTAB-6134 training cohort. In both training and external validation cohorts, its robustness was supported by the reproducibility of moderate predictive accuracy with the AUC values close to or exceeding 0.7. The superior accuracy of the 7-gene signature in both OS prediction and DFS prediction suggested that it could serve as a supplement to the existing staging system for prognosis evaluation and treatment decision. In addition to its prognostic value, the clinical implications of the 7-gene signature were also compelling.

Surgical resection combined with adjuvant chemotherapy has been the standard therapy for resectable PDAC patients (Von Hoff et al., 2013). Unfortunately, the clinical benefit response rates of chemotherapy are extremely low (Burris et al., 1997), and nonresponsive patients may experience a variety of adverse effects, including asthenia and nausea (Phua et al., 2018). Developing predictive biomarkers that can maximize survival benefits and minimize side effects is of significant importance for PDAC patients (Oshi et al., 2020). In this study, we found that the risk score was significantly related to chemotherapeutic sensitivity. In the cases who had received adjuvant chemotherapy, this signature could efficiently distinguish patients with different DFS time. We hypothesize that the 7-gene score may have a role in fostering personalized oncology to exempt PDAC patients from unnecessary cytotoxicity and heavy financial burden brought by overtreatment.

Recently, immunotherapy has drastically increased patient survival in multiple cancers, but it failed to elicit responses in the vast majority of patients with PDAC (Leinwand and Miller, 2020). The decreased number of tumor-infiltrating lymphocytes in a tumor microenvironment likely allows anticancer immunity to be overwhelmed in PDAC (Vonderheide and Bayne, 2013). In this study, CD8^+^ T cells were less infiltrated in high-risk patients, while M2 macrophages were more infiltrated in high-risk patients. CD8^+^ T cells recognize and kill the cancer cell (Farhood et al., 2019) and indicate a favorable prognosis in PDAC (Zhang et al., 2018; Carstens et al., 2017). M2 macrophages facilitate PDAC progression (Kurahara et al., 2011) and indicate an unfavorable prognosis in PDAC (McGuigan et al., 2021; Hu et al., 2016). These findings confirmed the hazardous role of this signature and suggested that it could be used to predict the immunotherapy response.

To preliminarily clarify the underlying mechanism of the risk signature–mediated poor prognosis, we investigated the biological function of this signature. Multiple oncogenic pathways that were strongly associated with tumor progression, chemotherapeutic resistance, and immune cell infiltration were enriched. Biological processes and pathways, including but not limited to the PI3K-AKT signaling pathway, HIF-1 signaling pathway, and glycolysis, were all implicated in the regulation of malignant behavior and anticancer immunity (Xu et al., 2021b; Kobayashi et al., 2020; Sun and Meng, 2020; You et al., 2020). These findings could partly explain how the risk score affected patient survival and immune cell infiltration.

Despite improved OS prediction and DFS prediction compared with previous models (Kim et al., 2019; Feng et al., 2020), this study is still based on retrospective data and presents several limitations. First, the clinical utility of the 7-gene signature in PDAC management should be reviewed and determined in more prospective studies. Second, all cohorts used in the current study were relatively small, probably because of the low surgical resection rates of PDAC. Thus, it needs to be validated in larger cohorts. Third, further *in vivo* and *in vitro* experiments are needed in order to clarify the biological roles of seven genes in PDAC tumorigenesis. Finally, due to the lack of significant data, we are unable to adequately assess the relationship between seven gene expression and clinical feature. With the development of follow-up studies, we hope to supplement them in future studies.

In conclusion, we proposed a 7-gene signature that provided improved OS prediction and DFS prediction. The clinical implications and biological relevance of this signature have been completed explored. However, the predictive efficacy of this signature needs to be tested in more larger cohorts and prospective studies.

## Data Availability

Publicly available datasets were analyzed in this study. These data can be found here: Expression profiles and clinical data of the MTAB-6134 cohort and MTAB-6690 cohort were downloaded from the ArrayExpress database (https://www.ebi.ac.uk/arrayexpress/). Normalized RNA-sequencing (RNA-seq) data and clinical data of the PACA-CA cohort were retrieved and downloaded from the International Cancer Genome Consortium (ICGC, https://icgc.org/) database. Processed RNA-seq data, clinical information, and somatic mutation data of the TCGA cohort were obtained from the TCGA hub at UCSC Xena (https://tcga.xenahubs.net).

## Abbreviations

AUC, area under the curve; BP, biological process; DFS, disease-free survival; GO, Gene Ontology; ICGC, International Cancer Genome Consortium; KEGG, Kyoto Encyclopedia of Genes and Genomes; K-M, Kaplan–Meier; OS, overall survival; PDAC, pancreatic ductal adenocarcinoma; ROC, receiver operating characteristic; TCGA, the Cancer Genome Atlas; TNM, tumor, node, and metastasis.

## References

[B1] Ahluwalia P. Kolhe R. Gahlay G. K. (2021). The clinical relevance of gene expression based prognostic signatures in colorectal cancer. Biochimica et Biophysica Acta (BBA) - Reviews on Cancer 1875, 188513. 10.1016/j.bbcan.2021.188513 33493614

[B2] Ansari D. Bauden M. Bergström S. Rylance R. Marko-Varga G. Andersson R. (2017). Relationship between tumour size and outcome in pancreatic ductal adenocarcinoma. Br J Surg 104, 600–607. 10.1002/bjs.10471 28177521

[B3] Burris H. A. 3rd. Moore M. J. Andersen J. Green M. R. Rothenberg M. L. Modiano M. R. (1997). Improvements in survival and clinical benefit with gemcitabine as first-line therapy for patients with advanced pancreas cancer: a randomized trial. Jco 15, 2403–2413. 10.1200/jco.1997.15.6.2403 9196156

[B4] Carstens J. L. Correa de Sampaio P. Yang D. Barua S. Wang H. Rao A. (2017). Spatial computation of intratumoral T cells correlates with survival of patients with pancreatic cancer. Nat Commun 8, 15095. 10.1038/ncomms15095 28447602 PMC5414182

[B5] Chen B. Khodadoust M. S. Liu C. L. Newman A. M. Alizadeh A. A. (2018). Profiling Tumor Infiltrating Immune Cells with CIBERSORT. Methods Mol Biol 1711, 243–259. 10.1007/978-1-4939-7493-1_12 29344893 PMC5895181

[B6] De La Cruz MS Young AP Ruffin MT (2014). Diagnosis and management of pancreatic cancer. Am Fam Physician 89, 626–32. 24784121

[B7] Doultsinos D. Mills I. G. (2021). Derivation and Application of Molecular Signatures to Prostate Cancer: Opportunities and Challenges. Cancers 13, 495. 10.3390/cancers13030495 33525365 PMC7865812

[B8] Ducreux M. Cuhna A. S. Caramella C. Hollebecque A. Burtin P. Goéré D. (2015). Cancer of the pancreas: ESMO Clinical Practice Guidelines for diagnosis, treatment and follow-up. Annals of Oncology 26 (Suppl 5), v56–v68. 10.1093/annonc/mdv295 26314780

[B9] Farhood B. Najafi M. Mortezaee K. (2019). CD8+cytotoxic T lymphocytes in cancer immunotherapy: A review. J Cell Physiol 234, 8509–8521. 10.1002/jcp.27782 30520029

[B10] Feng Z. Shi M. Li K. Ma Y. Jiang L. Chen H. (2020). Development and validation of a cancer stem cell-related signature for prognostic prediction in pancreatic ductal adenocarcinoma. J Transl Med 18, 360. 10.1186/s12967-020-02527-1 32958051 PMC7507616

[B11] Garrido-Laguna I. Hidalgo M. (2015). Pancreatic cancer: from state-of-the-art treatments to promising novel therapies. Nat Rev Clin Oncol 12, 319–334. 10.1038/nrclinonc.2015.53 25824606

[B12] Ghaneh P. Kleeff J. Halloran C. M. Raraty M. Jackson R. Melling J. (2019). The Impact of Positive Resection Margins on Survival and Recurrence Following Resection and Adjuvant Chemotherapy for Pancreatic Ductal Adenocarcinoma. Ann Surg 269, 520–529. 10.1097/sla.0000000000002557 29068800

[B13] Gillen S. Schuster T. Meyer zum Büschenfelde C. Friess H. Kleeff J. (2010). Preoperative/neoadjuvant therapy in pancreatic cancer: a systematic review and meta-analysis of response and resection percentages. PLoS Med 7, e1000267. 10.1371/journal.pmed.1000267 20422030 PMC2857873

[B14] Gu M. Sun J. Zhang S. Chen J. Wang G. Ju S. (2021). A novel methylation signature predicts inferior outcome of patients with PDAC. Aging 13, 2851–2863. 10.18632/aging.202347 33550277 PMC7880369

[B15] Hu H. Hang J.-J. Han T. Zhuo M. Jiao F. Wang L.-W. (2016). The M2 phenotype of tumor-associated macrophages in the stroma confers a poor prognosis in pancreatic cancer. Tumor Biol. 37, 8657–8664. 10.1007/s13277-015-4741-z 26738860

[B16] Huang D. W. Sherman B. T. Tan Q. Kir J. Liu D. Bryant D. (2007). DAVID Bioinformatics Resources: expanded annotation database and novel algorithms to better extract biology from large gene lists. Nucleic Acids Res 35, W169–W175. 10.1093/nar/gkm415 17576678 PMC1933169

[B17] Huang J. Lok V. Ngai C. H. Zhang L. Yuan J. Lao X. Q. (2021). Worldwide Burden of, Risk Factors for, and Trends in Pancreatic Cancer. Gastroenterology 160, 744–754. 10.1053/j.gastro.2020.10.007 33058868

[B18] Juiz N. A. Iovanna J. Dusetti N. (2019). Pancreatic Cancer Heterogeneity Can Be Explained Beyond the Genome. Front. Oncol. 9, 246. 10.3389/fonc.2019.00246 31024848 PMC6460948

[B19] Kamisawa T. Wood L. D. Itoi T. Takaori K. (2016). Pancreatic cancer. The Lancet 388, 73–85. 10.1016/s0140-6736(16)00141-0 26830752

[B20] Kim J. Jo YH. Jang M. Nguyen NNY. Yun HR. Ko SH (2019). PAC-5 Gene Expression Signature for Predicting Prognosis of Patients with Pancreatic Adenocarcinoma. Cancers 11, 1749. 10.3390/cancers11111749 PMC6896100 31703415

[B21] Kleeff J. Korc M. Apte M. La Vecchia C. Johnson C. D. Biankin A. V. (2016). Pancreatic cancer. Nat Rev Dis Primers 2, 16022. 10.1038/nrdp.2016.22 27158978

[B22] Kobayashi Y. Lim S.-O. Yamaguchi H. (2020). Oncogenic signaling pathways associated with immune evasion and resistance to immune checkpoint inhibitors in cancer. Seminars in Cancer Biology 65, 51–64. 10.1016/j.semcancer.2019.11.011 31874279

[B23] Kurahara H. Shinchi H. Mataki Y. Maemura K. Noma H. Kubo F. (2011). Significance of M2-polarized tumor-associated macrophage in pancreatic cancer. Journal of Surgical Research 167, e211–e219. 10.1016/j.jss.2009.05.026 19765725

[B24] Leinwand J. Miller G. (2020). Regulation and modulation of antitumor immunity in pancreatic cancer. Nat Immunol 21, 1152–1159. 10.1038/s41590-020-0761-y 32807942

[B25] Luo L Li Y Huang C Lin Y Su Y Cen H (2021). A new 7-gene survival score assay for pancreatic cancer patient prognosis prediction. Am J Cancer Res 11, 495–512. 33575083 PMC7868749

[B26] Macías N. Sayagués J. M. Esteban C. Iglesias M. González L. M. Quiñones-Sampedro J. (2018). Histologic Tumor Grade and Preoperative Bilary Drainage are the Unique Independent Prognostic Factors of Survival in Pancreatic Ductal Adenocarcinoma Patients After Pancreaticoduodenectomy. J Clin Gastroenterol 52, e11–e17. 10.1097/mcg.0000000000000793 28059940

[B27] Maeda S. Moore A. M. Yohanathan L. Hata T. Truty M. J. Smoot R. L. (2020). Impact of resection margin status on survival in pancreatic cancer patients after neoadjuvant treatment and pancreatoduodenectomy. Surgery 167, 803–811. 10.1016/j.surg.2019.12.008 31992444

[B28] McGranahan N. Swanton C. (2017). Clonal Heterogeneity and Tumor Evolution: Past, Present, and the Future. Cell 168, 613–628. 10.1016/j.cell.2017.01.018 28187284

[B29] McGuigan A. J. Coleman H. G. McCain R. S. Kelly P. J. Johnston D. I. Taylor M. A. (2021). Immune cell infiltrates as prognostic biomarkers in pancreatic ductal adenocarcinoma: a systematic review and meta‐analysis. J Pathol Clin Res 7, 99–112. 10.1002/cjp2.192 33481339 PMC7869931

[B30] Oshi M. Tokumaru Y. Patel A. Yan L. Matsuyama R. Endo I. (2020). A Novel Four-Gene Score to Predict Pathologically Complete (R0) Resection and Survival in Pancreatic Cancer. Cancers 12, 3635. 10.3390/cancers12123635 PMC7761977 33291601

[B31] Phua L. C. Goh S. Tai D. W. M. Leow W. Q. Alkaff S. M. F. Chan C. Y. (2018). Metabolomic prediction of treatment outcome in pancreatic ductal adenocarcinoma patients receiving gemcitabine. Cancer Chemother Pharmacol 81, 277–289. 10.1007/s00280-017-3475-6 29196965

[B32] Sho M. Murakami Y. Motoi F. Satoi S. Matsumoto I. Kawai M. (2015). Postoperative prognosis of pancreatic cancer with para-aortic lymph node metastasis: a multicenter study on 822 patients. J Gastroenterol 50, 694–702. 10.1007/s00535-014-1005-4 25341657

[B33] Siegel R. L. Miller K. D. Fuchs H. E. Jemal A. (2021). Cancer Statistics, 2021. CA A Cancer J. Clin. 71, 7–33. 10.3322/caac.21654 33433946

[B34] Strobel O. Hank T. Hinz U. Bergmann F. Schneider L. Springfeld C. (2017). Pancreatic Cancer Surgery. Ann Surg 265, 565–573. 10.1097/sla.0000000000001731 27918310

[B35] Sun P. Meng L.-h. (2020). Emerging roles of class I PI3K inhibitors in modulating tumor microenvironment and immunity. Acta Pharmacol Sin 41, 1395–1402. 10.1038/s41401-020-00500-8 32939035 PMC7656798

[B36] Supplitt S. Karpinski P. Sasiadek M. Laczmanska I. (2021). Current Achievements and Applications of Transcriptomics in Personalized Cancer Medicine. Ijms 22, 1422. 10.3390/ijms22031422 33572595 PMC7866970

[B37] Tummers W. S. Groen J. V. Sibinga Mulder B. G. Farina-Sarasqueta A. Morreau J. Putter H. (2019). Impact of resection margin status on recurrence and survival in pancreatic cancer surgery. Br J Surg 106, 1055–1065. 10.1002/bjs.11115 30883699 PMC6617755

[B38] van Roessel S. Kasumova G. G. Verheij J. Najarian R. M. Maggino L. de Pastena M. (2018). International Validation of the Eighth Edition of the American Joint Committee on Cancer (AJCC) TNM Staging System in Patients With Resected Pancreatic Cancer. JAMA Surg 153, e183617. 10.1001/jamasurg.2018.3617 30285076 PMC6583013

[B39] Von Hoff D. D. Ervin T. Arena F. P. Chiorean E. G. Infante J. Moore M. (2013). Increased survival in pancreatic cancer with nab-paclitaxel plus gemcitabine. N Engl J Med 369, 1691–1703. 10.1056/NEJMoa1304369 24131140 PMC4631139

[B40] Vonderheide R. H. Bayne L. J. (2013). Inflammatory networks and immune surveillance of pancreatic carcinoma. Current Opinion in Immunology 25, 200–205. 10.1016/j.coi.2013.01.006 23422836 PMC3647365

[B41] Xu D. Wang Y. Liu X. Zhou K. Wu J. Chen J. (2021a). Development and clinical validation of a novel 9-gene prognostic model based on multi-omics in pancreatic adenocarcinoma. Pharmacological Research 164, 105370. 10.1016/j.phrs.2020.105370 33316381

[B42] Xu K. Yin N. Peng M. Stamatiades E. G. Shyu A. Li P. (2021b). Glycolysis fuels phosphoinositide 3-kinase signaling to bolster T cell immunity. Science 371, 405–410. 10.1126/science.abb2683 33479154 PMC8380312

[B43] You L. Wu W. Wang X. Fang L. Adam V. Nepovimova E. (2020). The role of hypoxia‐inducible factor 1 in tumor immune evasion. Med Res Rev 41, 1622–1643. 10.1002/med.21771 33305856

[B44] Yu F. Quan F. Xu J. Zhang Y. Xie Y. Zhang J. (2019). Breast cancer prognosis signature: linking risk stratification to disease subtypes. Brief Bioinform 20, 2130–2140. 10.1093/bib/bby073 30184043

[B45] Zhang J. Wang Y. F. Wu B. Zhong Z. X. Wang K. X. Yang L. Q. (2018). Intraepithelial Attack Rather than Intratumorally Infiltration of CD8+T Lymphocytes is a Favorable Prognostic Indicator in Pancreatic Ductal Adenocarcinoma. Cmm 17, 689–698. 10.2174/1566524018666180308115705 PMC6416191 29521231

[B46] Zheng L. Xue J. Jaffee E. M. Habtezion A. (2013). Role of immune cells and immune-based therapies in pancreatitis and pancreatic ductal adenocarcinoma. Gastroenterology 144, 1230–1240. 10.1053/j.gastro.2012.12.042 23622132 PMC3641650

