# Supplementary figures and images for: Development and Validation of a 7-Gene Prognostic Signature to Improve Survival Prediction in Pancreatic Ductal Adenocarcinoma
Source: Front Mol Biosci. 2021 May 21;8:676291. doi: 10.3389/fmolb.2021.676291 (PMC8176016; doi:10.3389/fmolb.2021.676291)

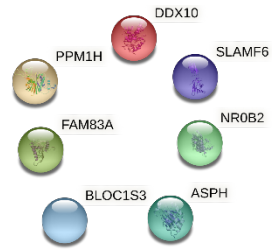

**Supplementary Figure S1. Protein-protein interaction network of the seven genes.**

Supplement: Supplementary file 1 [file DataSheet1.PDF]
